# Target Enrichment Improves Mapping of Complex Traits by Deep Sequencing

**DOI:** 10.1534/g3.115.023671

**Published:** 2015-10-30

**Authors:** Jianjun Guo, Jue Fan, Bernard A. Hauser, Seung Y. Rhee

**Affiliations:** * Department of Plant Biology, Carnegie Institution for Science, Stanford, California 94305; † Department of Biology, Plant Molecular and Cellular Biology Program, University of Florida, Gainesville, Florida 32611

**Keywords:** target-enriched extreme quantitative trait locus mapping, bulked segregant analysis, *Arabidopsis thaliana*, seed size, salt tolerance

## Abstract

Complex traits such as crop performance and human diseases are controlled by multiple genetic loci, many of which have small effects and often go undetected by traditional quantitative trait locus (QTL) mapping. Recently, bulked segregant analysis with large F2 pools and genome-level markers (named extreme-QTL or X-QTL mapping) has been used to identify many QTL. To estimate parameters impacting QTL detection for X-QTL mapping, we simulated the effects of population size, marker density, and sequencing depth of markers on QTL detectability for traits with differing heritabilities. These simulations indicate that a high (>90%) chance of detecting QTL with at least 5% effect requires 5000× sequencing depth for a trait with heritability of 0.4−0.7. For most eukaryotic organisms, whole-genome sequencing at this depth is not economically feasible. Therefore, we tested and confirmed the feasibility of applying deep sequencing of target-enriched markers for X-QTL mapping. We used two traits in *Arabidopsis thaliana* with different heritabilities: seed size (H^2^ = 0.61) and seedling greening in response to salt (H^2^ = 0.94). We used a modified G test to identify QTL regions and developed a model-based statistical framework to resolve individual peaks by incorporating recombination rates. Multiple QTL were identified for both traits, including previously undiscovered QTL. We call our method target-enriched X-QTL (TEX-QTL) mapping; this mapping approach is not limited by the genome size or the availability of recombinant inbred populations and should be applicable to many organisms and traits.

Phenotypic variation of complex traits such as crop performance and human diseases are controlled by multiple genetic loci, many with small effect size (Buckler *et al.* 2009; Plomin *et al.* 2009; Peiffer *et al.* 2014). Identification of all the genetic loci contributing to the phenotypic variation of a trait is an important step toward understanding the underlying molecular mechanisms of trait evolution. Quantitative trait locus (QTL) mapping and genome-wide association studies (GWAS) are two common approaches for dissecting the genetic landscape of complex traits. Compared with QTL mapping, GWAS usually have greater mapping resolution, but the underlying population structure can cause many false-positives (Korte and Farlow 2013). QTL mapping using populations generated from controlled crosses typically uses small populations (a few hundred), which can lead to not only an underestimation of the number of QTL but also an overestimation of the contributions of the detected QTL to the observed phenotypic variation (Melchinger *et al.* 2004). To increase the detection capacity of the genetic loci underlying complex traits with QTL mapping, a large population is required to increase both statistical power and recombination events (Ehrenreich *et al.* 2010; Mackay *et al.* 2009). This is rarely done because genotyping and phenotyping a large number of individuals is costly and labor intensive.

Bulked segregant analysis was developed as a fast approach to map major QTL, where individuals with phenotypes at two extremes are pooled separately and genetic markers from each pool are compared to determine the linkage between the markers and the phenotype (Michelmore *et al.* 1991). Recently, bulked segregant analysis with large F2 pools and genome-level single-nucleotide polymorphism (SNP) markers (also named extreme-QTL or X-QTL) has been applied in yeast and *Drosophila melanogaster* (Lai *et al.* 2007; Ehrenreich *et al.* 2010). This approach demonstrated the power of X-QTL mapping to dissect multiple loci contributing to complex traits. A large population size (Ehrenreich *et al.* 2010) and 10–20% bulk size (Magwene *et al.* 2011) are necessary for increasing the detection power of X-QTL mapping. In addition, high sequencing depth was predicted to be necessary for increased QTL detectability (Magwene *et al.* 2011). However, a comprehensive power analysis for X-QTL mapping that considers all the major contributing factors for increasing QTL detection power has not been conducted.

Here, we performed an in-depth power analysis to guide the choices of experimental conditions for increased QTL detection using X-QTL mapping. The simulations suggested that both a large population size and high sequencing depth of markers are needed to increase the detection power of X-QTL mapping. To achieve the required sequencing depth at a reasonable cost, we used target enrichment to sequence SNP markers and developed a statistical framework based on model selection to estimate intervals of mapped QTL peak locations. We call this mapping approach target-enriched X-QTL, or TEX-QTL mapping. In *Arabidopsis thaliana*, we applied TEX-QTL mapping to identify QTL for two traits: 1) seedling greening in response to salt and 2) seed size. Using both theoretical and empirical approaches, we demonstrated increased QTL detection power of TEX-QTL mapping.

## Materials and Methods

### TEX-QTL detectability simulations

To identify parameters that could increase QTL detection power using TEX-QTL mapping, we wrote an R script (Supporting Information, File S1) to perform simulations using the R (R Core Team 2013) package hypred (Technow 2013). The genome was set up as five chromosomes of 100 cM each. We chose additive QTL with no interactions and each chromosome contained one QTL. On the basis of experimental data in the literature, the spontaneous mutation rate was set to 7.0E-9 (Ossowski *et al.* 2010). Phenotypic values were assigned as the sum of genotypic and random environmental effects (normally distributed). The maximal size of the environmental effect was determined based on the heritability.

Two experimental designs were simulated: high *vs.* control bulks (one-tailed bulk design) and high *vs.* low bulks (two-tailed bulk design). We randomly selected 10% of the individuals as the control bulk, and top/bottom 10% of the individuals with the highest/lowest phenotypic values as the high/low bulks. G-test was performed to detect markers with a significant difference in allele frequency between the two bulks at the significance level of 0.01. The p-value was corrected for multiple testing by the Benjamini-Hochberg method (Benjamini and Hochberg 1995). Under a fixed set of parameters (QTL effect size, population size, marker density, and sequencing depth), the probability of QTL detection was determined based on 100 simulations for each of 10 heritability values ranging from 0 to 1. To simulate a QTL with 20% effect, five equally contributing QTL for the five chromosomes were used. To simulate QTL with 10% and 5% effects, five QTL with effect sizes of 70%, 10%, 10%, 5%, and 5% were used.

To test the effect of population size, marker density was set to 5 per cM. Population sizes of 200, 1000, 4000 and 10,000 were simulated by randomly selecting individuals from the two bulks. To test the effect of sequencing depth, population size was fixed to 10,000 and marker density was set to five markers per cM. Sequencing depths of 100×, 500×, 1000×, and 5000× were selected for simulations. Finally, to test the effect of marker density, population size was fixed to 10,000 and sequencing depth was fixed to 1000×. Marker densities of 0.2, 1, 5, and 20 per cM were compared in the simulations.

### Bulked segregant analysis

For the seed size trait analysis, plants were grown in a greenhouse with 16-hr/8-hr light/dark cycle at 22°. Reciprocal crosses between Sha (ABRCstock number: CS22652) and Tsu-1 (ABRC stock number: CS22641) parents were made. F2 seeds were separated by size with sieves from Industrial Netting (Minneapolis, MN). Sieves with pore sizes of 200, 250, 300, and 400 µm separated 140,000 F2 seeds into four discrete bins: 1) <200 μm, 2) 200–250 μm, 3) 250–300 μm, and 4) 300–400 μm. Most seeds sorted into the 250–300 μm bin. The high bulk had 10,000 seeds from the 300- to 400-µm bin, which made up 7.1% of the population. While ~1% of the seeds were smaller than 200 μm, a lot of chaff, debris, and broken seeds were in this bin. Therefore, this bin was not used for further analysis. The 200- to 250-µm bin contained the bottom 14% of the population. From this bin, 10,000 seeds were selected randomly to form the low bulk. A Mettler MT5 microbalance was used to measure seed mass. To measure seed dimensions, captured stereomicroscope images were analyzed using the Bisque Seed Size tool (Kvilekval *et al.* 2010). Assuming *Arabidopsis* seeds form an ellipsoid, their volume was estimated using the formula: 4/3π(w*/*2)^2^(l*/*2) (where w = seed width, l = seed length). Seeds from each bulk were grown on media (1/2 × Murashige and Skoog (MS), 0.05% 2-(*N*-morpholino)ethanesulfonic acid, 3% sucrose, 0.7% agar, pH 5.7) for 5 d, and seedlings were pooled for genomic DNA extraction and target enrichment.

For salt tolerance bulked segregant analysis, 16,000 F2 individuals descended from crossing Sha female (ABRC stock number: CS22652) to Tsu-1 male (ABRC stock number: CS22641) were planted on growth media (1/2 × MS, 0.05% 2-(*N*-morpholino)ethanesulfonic acid, 3% sucrose, 0.7% agar, pH 5.7) containing 0 or 150 mM NaCl. Approximately 2000 seeds were planted on each 150 × 15-mm Petri dish. Plants were grown in an environmentally controlled chamber with 16-hr/8-hr light/dark cycle at 22°. A sample plate (60 × 15 mm) with approximately 200 seeds was used to estimate the proportion of green seedlings. The difference in seed density between the sample plate (7 seeds per cm^2^) and the bulk-selection plates (11.3 seeds per cm^2^) did not significantly affect the timing of seedling greening on salt plates under these growth conditions (data not shown). Sixteen-thousand F2 individuals were germinated on agar plates containing 150 mM NaCl, and the seedlings with green cotyledons were harvested between 96- to 120-hr after germination; these seedlings accounted for 10% of the population and formed the high bulk (“salt-tolerant bulk”). For the control bulk, we randomly selected 10% of seedlings from 16,000 F2 plants grown on agar plates without NaCl 72 hr after germination. We used the control bulk rather than the 10% most sensitive seedlings for comparison because poor germination on high salt could arise from many reasons not directly related to salt stress response. In total, three independent experiments were conducted.

### Determining heritability

To determine the broad-sense heritability, the environmental and total phenotypic variances were estimated by the mean within-accession and between-accession variances of Sha and Tsu-1 (Griffiths *et al.* 2000). The heritability of seed size was estimated from 20 seeds per accession (File S2) and that of seedling greening in response to salt was estimated from data collected from four independent experiments with 25−59 seedlings per accession per experiment (File S3). For seed size, the parental and F1 variances were used to measure environmental variance.

### Probe design

We used the “SNPs between accessions” tool provided on the Polymorph Web site (Clark *et al.* 2007) to search for SNPs between Sha and Tsu-1 genome using the TAIR7 *Arabidopsis* genome (www.arabidopsis.org) as the reference genome. Because the sequence from only one accession can be used to design each probe, we positioned probes at regions that are one or two nucleotide(s) away from the targeted SNP positions to minimize capture bias. These sequences (File S4) were sent to Agilent for the synthesis of biotinylated-RNA probes for target enrichment.

### DNA sample preparation

For each pooled tissue sample, genomic DNA was isolated with the QIAGEN plant genomic DNA maxi prep kit. To remove polysaccharide contamination, the isolated genomic DNA was re-extracted with a cetyltrimethyl ammonium bromide (CTAB) concentration. For each sample, 3 µg of genomic DNA was fragmented with a Covaris S220 Focused-ultrasonicator. The library preparation and probe hybridization were conducted following the protocol of Agilent SureSelect target enrichment system (Agilent 2013). To summarize briefly, fragmented genomic DNA was end-repaired with a single adenine nucleotide overhang and ligated to sequencing adaptors with a single thymine nucleotide overhang. The successfully ligated DNA fragments were amplified by polymerase chain reaction and hybridized with biotin-labeled RNA probes. The RNA/DNA hybrids were isolated by incubation with streptavidin-coated magnetic beads, which bound the biotin. The enriched genomic DNA fragments were released, bar-coded, and sequenced using Illumina’s HiSeq with 100 base, paired-end sequencing (National Center for Biotechnology Information accession number: SRX659684).

### TEX-QTL mapping

Paired-end reads were aligned to the TAIR10 (Lamesch *et al.* 2012) reference genome with the use of Bowtie2 (Langmead and Salzberg 2012). Mapped reads were reformatted with the mpileup function of SAMtools (Li *et al.* 2009), and the allele frequencies at polymorphic sites with Phred quality score of >20 and coverage >500 were counted by varScan2 (Koboldt *et al.* 2012).

The G-test was used to detect the SNP markers whose Sha/Tsu-1 ratio between two bulk populations significantly differed. To reduce the random binomial sampling noise of two alleles introduced by sequencing, the smoothed G test (G′ test) (Magwene *et al.* 2011) was performed at each SNP marker position with a sliding window of 9 Mb (∼36 cM as determined in *Results* section) and the tri-cube weight function (Cleveland 1979).

### Simulations to determine the smoothing window size of TEX-QTL

To investigate the effect of G′-test’s smoothing window sizes on the number of false-positive and false-negative peaks, we simulated TEX-QTL on a chromosome with one QTL by using a range of smoothing window sizes. One hundred simulations were carried out for each window size. The length of the chromosome was set to 100 cM. The single QTL was positioned in the middle and flanked by 50 cM on both sides. We used the high *vs.* control bulk design and the beneficial allele in the high bulk was set to 0.6. The sequencing coverage was set to 5000×. The marker density was set to 5 per cM, which was used in our experimental design. The tested smoothing window sizes were 16, 20, 24, 28, 32, and 36 cM.

We then applied TEX-QTL on a chromosome with two QTL using 36 cM as the smoothing window size, chosen based on the results of the 1-QTL simulations mentioned previously. Two QTL were set to have the same allele frequency of 0.6. Various distances between the two QTL were simulated to determine the probability of two QTL merging into one when their distance was smaller than the smoothing window size. The distances of 8, 12, 16, 20, 24, 28, and 32 cM were compared. The other parameters were the same as the single QTL simulations.

### Data availability

Code used to generate the simulated data is provided in File S1. File S2 contains seed dimension data. File S3 contains green seedling response to salt heritability data. File S4 contains all probe sequences and genomic locations. File S5 contains seed weight data. File S6 contains the TEX-QTL mapping program in Python. File S7 contains detailed descriptions for generating the expected shape of G and G′ for linked causal SNPs. Sequence data are available at GenBank with the accession number: SRX659684.

## Results

### Determining TEX-QTL mapping parameters using power analysis

To increase the detection power of TEX-QTL mapping, we tested parameters that can influence QTL detectability such as population size, sequencing depth, marker density, heritability, and QTL effect size using simulations. The bulk size was not simulated because a previous simulation study showed that the bulk size of 10–20% is needed for increased QTL detection power (Magwene *et al.* 2011). We simulated two commonly used bulk-selection schemes, two-tailed (subpopulations with phenotypes at the two extreme ends of the phenotypic distribution) and one-tailed (one subpopulation at one end of the phenotypic distribution and a random selection of equal-size bulk from the population). The one-tailed design is used where individuals from one end of the phenotypic distribution cannot be effectively collected (*e.g.*, cell survival experiments) (Ehrenreich *et al.* 2010).

**Figure 1:**
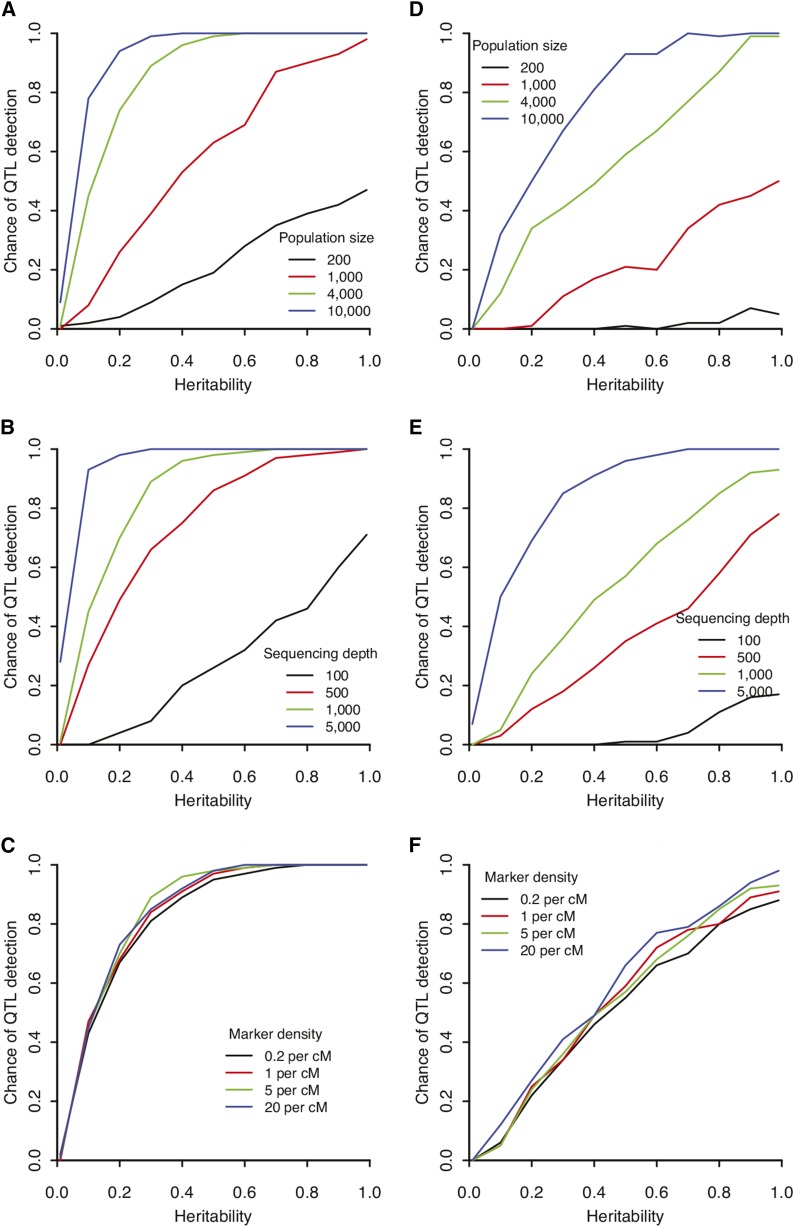
Simulations revealing the probability of finding a quantitative trait locus (QTL) with 5% effect for two target-enriched extreme QTL mapping designs: two-tailed (A−C) and one-tailed (D−F). (A) and (D) Effect of population size with individual genotyping. Marker density was set to 5 per cM. (B) and (E) Effect of sequencing depth. The population size was fixed to 10,000, and the marker density was set to five markers per cM. (C) and (F) Effect of marker density. The population size and sequencing depth are fixed to 10,000 and 1000, respectively. For each combination of parameters, 100 simulations were conducted.

Our simulation results indicate that the detection power was positively correlated with heritability, population size, and sequencing depth (Figure 1). In addition, the one-tailed bulk design was less powerful than the two-tailed bulk design at comparable population size, sequencing depth, and heritability (Figure 1). For traits with greater heritability (>0.7), an F2 population size of 4000 is sufficient to detect all additive QTL of greater than 5% effect size when the two-tailed bulk design was used (Figure 1A), whereas a population size of 10,000 is needed to achieve the same detection power with one-tailed bulk design (Figure 1D). If we consider the difference in the effect of the sequencing depth, the two-tailed bulk design needs 1000× per marker for >99% chance of detecting a 5%-effect QTL, whereas the one-tailed bulk design needs a sequencing depth of 5000× (Figure 1, B and E) to reach equivalent detection power. Similar trends were observed for traits with moderate heritability (0.4−0.7). For example, 5000× sequencing depth is sufficient for 99% chance of detecting a 5%-effect QTL with a two-tailed bulk design, but has the detection power of just 90% with one-tailed bulk design (Figure 1, B and E). In contrast, marker density (0.2−20 markers per cM) had little effect on the detectability of QTL under both designs (Figure 1, C and F). Our simulation of the effect of sequencing depth was conducted with the assumption of a large population size (>4000 F2 individuals) since a smaller population size would limit the QTL detection power as shown in Figure 1, A and D.

Similarly, for QTL of 10%- or 20%-effect sizes, greater detection power was observed in the two-tailed bulk design compared with that in the one-tailed bulk design when population size, sequencing depth and heritability were held equal (Figure S1). Moreover, larger population size, greater sequencing depth, and increased heritability all contributed to greater QTL detection power (Figure S1). In contrast, marker density did not have a significant effect on detectability of QTL at the range of 0.2−20 markers per cM (Figure S1C, F, I, and L). In addition, for QTL with larger effect sizes, a similar detection power could be achieved with smaller population size, lower marker sequencing depth, and lower heritability (Figure 1 and Figure S1).

### Developing TEX-QTL mapping strategy using two complex traits in *Arabidopsis* and different bulk-selection schemes

**Figure 2:**
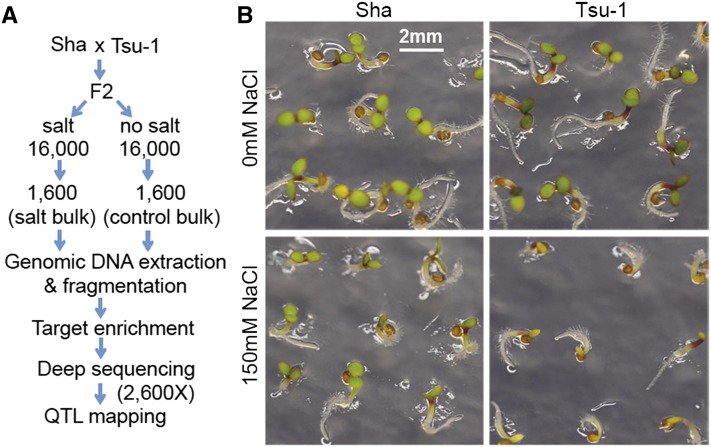
Identification of seedling greening in response to salt quantitative trait locus (QTL) by the use of target-enriched extreme QTL (TEX-QTL) mapping with F2 populations of Sha × Tsu-1 *Arabidopsis thaliana* ecotypes. (A) A schematic of the TEX-QTL mapping procedure. (B) Differential seedling greening of Sha and Tsu-1 parental lines grown on control or salt medium. Pictures were taken 4 d after germination.

Guided by the parameters determined from the simulations, we developed the TEX-QTL mapping method by using two complex traits in *Arabidopsis thaliana*: seed size with moderate (0.61) heritability and seedling greening in response to salt with high (0.94) heritability (Figure 2A, File S2, and File S3). The high *vs.* low bulk design was used to map seed size QTL and high *vs.* control design was used to map salt response QTL.

For the seed size trait, Tsu-1 seeds are significantly larger than Sha seeds (*P* < 0.001, one-way analysis of variance test) (Table 1, File S2). We screened 140,000 F2 seeds from Sha and Tsu-1 crosses and used the two-tailed bulk design to select top and bottom 7% as the high and low bulks. Interestingly, a significant maternal effect on seed mass was observed in F1 but not in F2 seeds (Table 1, File S5). When Tsu-1 was used as the female in crosses, the resulting F1 and F2 seeds were heavier than those seeds descended from crosses where Sha was the female. We therefore mapped QTL with F2 populations generated from reciprocal crosses.

**Table 1:** Mass and size of seeds and progenitors used for QTL analysis

Genotype	Seed Mass, µg N = 12	Seed Dimensions (length × width µm) N = 10 Unless Indicated
Sha (Parental)	18.5 ± 0.8	477 ± 6 × 269 ± 4 N = 20
Tsu-1 (Parental)	26.9 ± 0.8	512 ± 12 × 320 ± 8 N = 20
Sha* × Tsu-1 (F1)	28.5 ± 0.8	1090 ± 65 × 648 ± 32 N = 12
Tsu-1* × Sha (F1)	37.6 ± 0.6	1080 ± 22 × 686 ± 18 N = 12
Sha* × Tsu-1 (F2)	23.3 ± 1.1	505 ± 12 × 304 ± 6
Tsu-1* × Sha (F2)	25.8 ± 1.2	500 ± 9 × 311 ± 5
Sha* × Tsu-1 (F2) (upper bulk)	26.9 ± 1.2	513 ± 13 × 335 ± 6
Sha* × Tsu-1 (F2) (lower bulk)	18.0 ± 1.1	487 ± 14 × 280 ± 7
Tsu-1* × Sha (F2) (upper bulk)	31.0 ± 1.2	538 ± 9 × 343 ± 3
Tsu-1* × Sha (F2) (lower bulk)	19.1 ± 0.6	441 ± 12 × 275 ± 6

* Indicates the female parent in the cross. The mean ± SE is reported. N indicates sample size. QTL, quantitative trait locus.

For seedling greening in response to salt, a one-tailed bulk design was employed because of the difficulty in obtaining the salt sensitive bulk. We first screened 40 *Arabidopsis thaliana* accessions for their ability to germinate and establish green seedlings when grown with 150 mM NaCl in the media. The 40 accessions belong to the 64 core accessions collected world-wide representing a broad genetic diversity (McKhann *et al.* 2004). Among the 40 accessions tested, Sha and Tsu-1 displayed contrasting seedling greening phenotype in the presence of 150 mM NaCl (Figure 2B). We performed bulked segregant analysis by screening 16,000 F2 seeds derived from crossing Sha (female) and Tsu-1 (male). The high bulk consisted top 10% (1600) of seedlings with green cotyledons grown in 150 mM NaCl. The same number (1600) of seedlings from the control plate was selected as the control bulk.

### Marker selection and quality assessment

The power analysis showed that sequencing depth is more important than marker density for bulked segregant analysis with a large F2 population. We therefore incorporated the target enrichment technique, which initially was developed to reduce genome-sequencing costs by sequencing only exons (Summerer 2009), to enrich and sequence only selected SNPs to achieve high sequencing depth desired for increased QTL detection power.

We designed 110 bp probes against 2478 SNPs approximately evenly distributed across the genome. The probes were enriched and sequenced with Illumina HiSeq with an average sequencing depth of 2600× per probe for seedling greening in response to salt and 1610× per probe for seed size. Approximately 200 bp surrounding each probe was sequenced by paired-end sequencing, which resulted in the detection of 4587 SNP markers.

Since the allelic status of the probe can influence the frequency of captured allelic sequence at heterozygous loci (Asan *et al.* 2011), we assessed the allelic bias of probe capture and the potential impact of the capture bias on TEX-QTL mapping. To test this, we extracted genomic DNA from Sha × Tsu-1 F1 plants from three biological replicates and subjected it to target enrichment and sequencing at an average depth of 2600× from each replicate. If the capture is unbiased, the expected allele frequency is 0.5 for both Sha and Tsu-1 alleles. We observed a range of distribution for allelic frequency (Figure S2), indicating the presence of allelic bias during the capture process. However, more than 97% of the probes showed consistent allelic frequencies among the three independent F1 capture events (standard deviation < 0.05). We discarded the probes that showed allelic frequency and capture bias outside of the 95% confidence interval of the expected mean of 0.5 (Figure S2). A total of 2425 probes and 4278 SNP markers were retained for further analysis.

We next assessed the reproducibility of TEX-QTL mapping. Using three independent bulk selections of the salt response experiments, we calculated pair-wise correlations of the G-values of all the 4278 SNP markers between replicates. We observed good reproducibility between each pair of the replicates with R^2^ = 0.63-0.66 (Figure S3). Recently, a smoothed version of G test (G′ test) was developed to account for the variation introduced by bulk selection and sequencing of the bulks (Magwene *et al.* 2011). After we applied the G′ test, the correlation between each pair of the replicates improved to R^2^ = 0.90−0.94 (Figure S3).

Because of the high overall reproducibility between the replicates, we tested whether the data from the three biological replicates could be combined to increase sequencing depth of the markers using repeated G-tests (McDonald 2009). For most of the SNP markers (3903/4278 for salt response and 3158/4278 for seed size), we observed no significant heterogeneity among the replicates. After removing the markers showing significant deviations among the replicates, the data from the biological replicates were pooled to generate the final QTL maps, which had an average sequencing depth of 7800× for the salt response trait and 6440× for the seed size trait.

### Identification of statistically significant markers

The observed allele frequency of the markers deviates from the true allele frequency by two sources of variations: the sequencing coverage of the SNP markers and binominal sampling of two alleles in the bulk (Magwene *et al.* 2011), the latter being stochastic. Under the whole-genome sequencing scheme, the former is also stochastic, since the distribution of sequencing coverage is ideally Poisson (Lander and Waterman 1988). Therefore, it is possible to simultaneously remove both types of the random variations by kernel smoothing (Magwene *et al.* 2011). However, sequencing coverage of the SNP markers captured by target enrichment did not follow a Poisson distribution (Figure S4). The interquartile ranges (IQR) of the control bulk and high bulk of green seedling in response to salt are 5792 and 7105, respectively, indicating a highly nonuniform distribution compared to IQR = 119 for a Poisson distribution when the mean coverage is 7800×. Similarly, IQR of low and high bulk of seed size are 4321 and 4318, compared with IQR = 108 for a Poisson distribution when the mean coverage is 6440×.

To address the sequencing depth variation of the markers, we normalized them using a minimum threshold (set as 5000× for the salt response trait and 3000× for the seed size trait). The SNP markers with sequencing coverage higher than the threshold were downscaled proportionally to the threshold and those with lower coverage were discarded. After the normalization, 2653 and 2850 markers were retained for the salt response and seed size traits, respectively.

**Figure 3:**
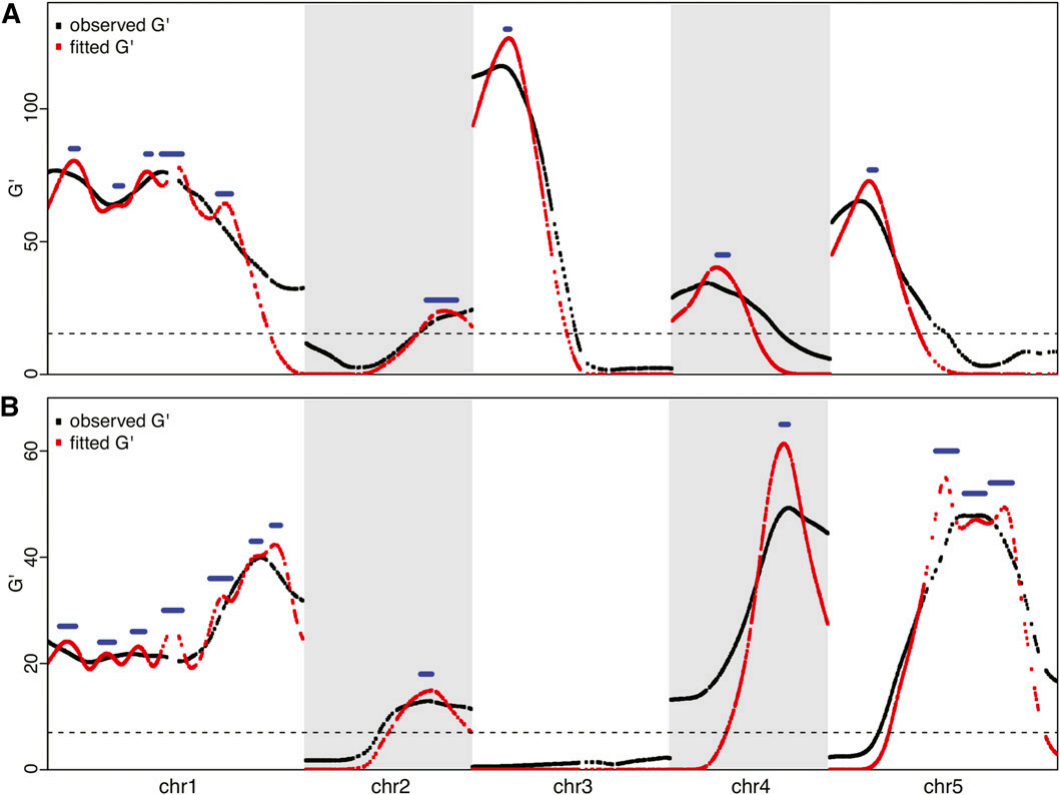
Mapping results using target-enriched extreme QTL. (A) Quantitative trait locus (QTL) map of *Arabidopsis thaliana* seedling greening in response to salt. (B) QTL map of *Arabidopsis thaliana* seed size. Black dots are observed G′ values of markers from three replicated experiments combined. The smoothing window of 36cM was used. Red dots are fitted G′ values based on the model-selection method. Blue bars show the support intervals of QTL peaks with 95% confidence. Dashed horizontal line shows the 1% false-discovery rate threshold of G′ values.

After normalizing for the sequencing depth, the binomial sampling variation was corrected by G′-test (Magwene *et al.* 2011). The G′ distributions of five chromosomes were plotted (Figure 3) and the false-discovery rate (FDR) was determined empirically. For each trait, the null distribution was calculated by performing G′ test among biological replicates. The distribution was then fitted to a log-normal distribution (Figure S5). Based on the fitted null distribution, SNP markers with FDR < 0.01 after we adjusted for multiple hypothesis testing were deemed significant (Benjamini and Hochberg 1995).

### Resolving QTL regions

Similar to a previous observation (Magwene *et al.* 2011), a large portion of the SNP markers have significant G′ values, due to the closely linked peaks on the same chromosome (Figure 3). To resolve linked QTL and estimate their location intervals, we developed a model selection approach to fit the observed G′ values based on a multiple-QTL model calculated from empirical recombination rates of *Arabidopsis* (Salomé *et al.* 2012).

#### Model selection:

To locate the regions with a high likelihood of containing the QTL, we developed a method to find the multiple-QTL model that best fits the observed G′ curve. Here we call the SNP marker that is closest to the causal site as the QTL marker. When only a single QTL is present in a chromosome, the G′ curve is expected to be unimodal (Magwene *et al.* 2011) and we can locate the SNP marker that has the maximal G′score as the QTL marker:

$$G^{\prime }\left(x\right)>G^{\prime }\left(i\right),\;\;for\;all\;i\;\neq x.$$

However, when multiple QTL are present in a chromosome, the allele frequency of each marker is influenced by genetic linkage to the flanking QTL, making the intuitive detection of the number and positions of the QTL difficult (Figure 3, black lines). Here we used model selection to fit a multiple-QTL model to the observed G′ values. Backward elimination strategy (Broman and Sen 2009) was applied to search the set of QTL positions that can best explain the observed G’ values of all the SNP markers.

A QTL model for each chromosome consists of QTL markers $\left(QTL_{1},\;QTL_{2},\ldots ,QTL_{m}\right)$ with their locations and allele frequencies $\left(p_{1},\;p_{2},\ldots ,p_{m}\right)$. The best model was selected using the following steps:

Step 1. The initial model included all the local maxima of the observed G′ curve as putative QTL markers. Local maximum was determined as a SNP marker *x* that has G′ value strictly higher than both of its flanking neighbors: $G^{\prime }\left(x\right)>G^{\prime }\left(x-1\right)$ and $G^{\prime }\left(x\right)>G^{\prime }\left(x+1\right)$. In addition, local minima of the absolute value of the first derivatives of G′ also were included as the putative QTL markers. The first derivative of a certain SNP marker was estimated by linear regression using SNP markers surrounding the site. The window size was the same as the one used in the G′-test. The one-to-one mappings of allele frequencies of the QTL markers to their G′ values were calculated based on the coverage, smoothing window of G′-test, and the bulk design. They were then used to map from G′ values to allele frequencies.
Step 2. The allele frequencies of all remaining markers were calculated based on the allele frequencies of the flanking putative QTL markers and recombination rates using the three-locus model assuming no interference from cross-overs (Liu 1997) (File S7). We used empirical recombination rates that were calculated based on crossover events averaged over a diverse set of F2 populations (Salomé *et al.* 2012). For markers between the ends of the chromosome and the QTL marker, two-locus model (Liu 1997) was applied conditioned on the allele frequency of the single flanking QTL marker. The computed allele frequencies of the non-QTL markers were used to derive estimated G′ scores of non-QTL markers.
Step 3. The estimated G′ curve was evaluated against the observed G′ curve using goodness of fit by root mean squared error (RMSE):
  $$RMSE=\sqrt{\frac{{\left(G^{\prime }{}_{i}-\hat{G^{\prime }{}_{i}}\right)}^{2}}{n}},$$

where *n* is the total number of SNP markers, $G^{\prime }{}_{i}$ is the observed G’ value of the *i*th SNP marker, and $\hat{G\prime_{i}}$ is the expected G’ value of the *i*th SNP marker.

Step 4. Step-wise backward elimination was used to determine whether any QTL marker in the current model should be omitted to increase the goodness of fit. The number of parameters in the model was penalized using Bayesian Information Criterion (BIC) (Schwarz 1978). Assuming that the deviations between the fitted model and observed G′ curve follow independent and identical normal distribution with a mean of zero, BIC could be written as:
  BIC=2n∗ln(RMSE)+2m∗ln(n),

where *n* is the total number of SNP markers and m is the number of QTL. If the BIC score decreased by removing a QTL marker, we updated the model by removing that marker. The backward elimination continued until BIC could not be improved any further.

Step 5. The positions and allele frequencies of the QTL markers were refined to improve the goodness of fit. Each QTL was chosen and adjusted for both its allele frequency and position while keeping all the other QTL markers fixed. The adjustment was iterated for all QTL markers until no changes in any of the parameters resulted in a better fit.
Step 6. We repeated steps 4 and 5 until no better model was found.

#### Estimating the interval of QTL location:

To identify regions with a high likelihood to contain QTL, we assumed the deviations between the fitted model and observed G′ curve are independently and identically distributed:

$$y_{i}=G^{\prime }{}_{i}-\hat{G^{\prime }{}_{i}}$$

$$y_{i}\;∼\;N\left(0,\;\sigma^{2}\right),$$

where $G^{\prime }{}_{i}$ and $\hat{G^{\prime }{}_{i}}$ are the observed and fitted G′ value of the SNP marker *i*, and *σ* is the standard deviation of $y_{i}.$ For random variable $y_{i},\;i=1,2,\ldots n$,

$$\frac{\sum_{i}{\left(y_{i}-0\right)}^{2}}{\sigma^{2}}$$

follows a $\chi^{2}$ distribution with n degrees of freedom, where n is the number of SNP markers. To rewrite the equation into the form of root mean square error (RMSE),

$$\frac{n*RMSE^{2}}{\sigma^{2}}$$

follows a $\chi^{2}$ distribution with n degrees of freedom. Using $\chi^{2}$probability distribution, we can define the 95% confidence interval of RMSE as:

$$P\left(\chi_{\frac{\alpha }{2},\;n}^{2}\leq \frac{n*RMSE^{2}}{\sigma^{2}}\leq \chi_{1-\frac{\alpha }{2},\;n}^{2}\right)=1-\alpha$$

where *α* is 95% here (and could be changed to other values as well). Rearranging the formula, we will get the interval of *σ* as:

$$\left[\sqrt{\frac{n}{\chi_{1-\frac{\alpha }{2},\;n}^{2}}}*RMSE,\sqrt{\frac{n}{\chi_{\frac{\alpha }{2},\;n}^{2}}}*RMSE\right]$$

Therefore, we estimated the confidence interval of each QTL peak to include all the nearby markers with smaller RMSE than the upper bound of the best model's 95% confidence interval when all the other parameters were fixed.

#### Window size determination:

The model selection method is sensitive to the smoothing window size of G′-test in two ways. First, smaller smoothing windows would leave more noise and potentially lead to false-positive peaks. On the other hand, larger smoothing windows would cause a decrease of resolution in peak identification because multiple peaks within the same smoothing window are likely to merge and become indistinguishable. Although a window size of 24–37 cM was suggested previously (Magwene *et al.* 2011), it is not clear what the best choice would be in terms of the model selection process. Here we used simulations to find the most appropriate window size to balance the number of false peaks with the possibility of merging closely lined peaks.

**Figure 4:**
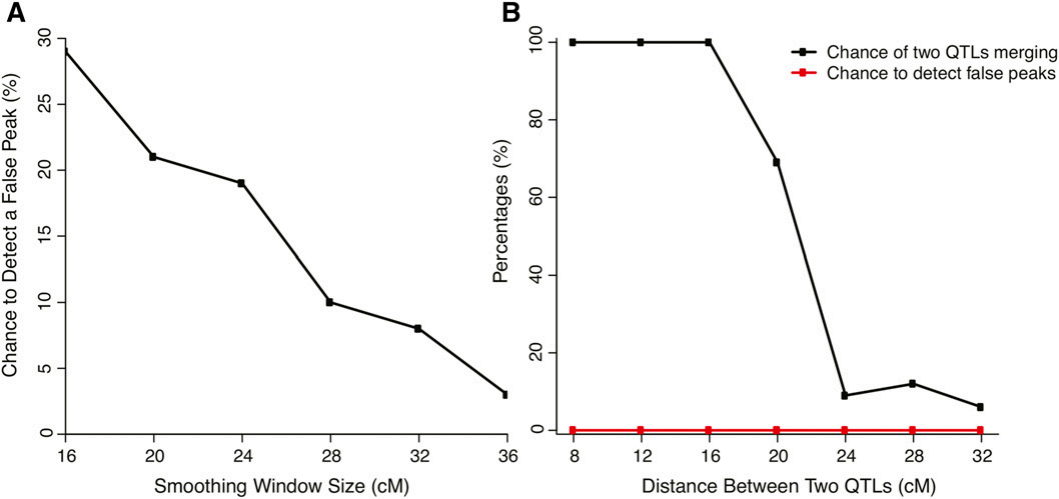
Assessing false-positive and false-negative rates of quantitative trait locus (QTL) detection by using simulations. (A) The probability of identifying false peaks for a 1-QTL chromosome using different smoothing window sizes. One hundred simulations were carried out for each data point. (B) The probability of missing true peaks and identifying false peaks for a two-QTL chromosome with various distances between the QTL. The two QTL have equal contribution to the phenotypic variation. The smoothing window is fixed at 36 cM. One hundred simulations were carried out for each data point.

To estimate the probability of identifying false peaks using different smoothing window sizes, we simulated a single QTL flanked by 50 cM on both sides. The probability of identifying false peaks was defined as the total number of false peaks found by the model selection process divided by the number of simulations, 100. As the window size increases, FDR decreased linearly (Figure 4A).

To estimate the detection limit of closely linked QTL, we simulated two equally contributing QTL linked on a 100 cM chromosome. We simulated the detectability of both QTL with various distances set between the two QTL. From the one-QTL simulations, we chose 36 cM as the smoothing window to minimize the false peaks. When the distance was less than 16 cM, the two QTL were not distinguishable and only one peak could be detected by TEX-QTL mapping (Figure 4B). When the distance was between 16–24 cM, the chance of merging decreased rapidly from 100 to less than 10%. When the distance was more than 24 cM, two peaks were most likely to be separable with the probability of less than 10%. We also tested the probability of detecting false peaks with the two-QTL simulations. They were 0 for all configurations, proving the window size of 36 cM is sufficient to minimize the noise of the G′ curve.

In summary, our simulations suggested that a larger window size (16−36 cM) decreases the chance of detecting false peaks. Our choice of 36 cM smoothing window achieves the lowest false detection rate within the range of our simulations. However, the limitation of our approach is the high chance of detecting two close-linked peaks (*e.g.*, two peaks with 20 cM or smaller distance) as a single merged peak.

### TEX-QTL mapping on two complex traits

Using TEX-QTL mapping, we identified several previously unidentified QTL as well as peaks that were previously reported (Table 2 and Table 3). For the greening in response to salt trait, the Sha allele increased salt tolerance for eight of nine QTL (GRS1−7, GRS9). The Tsu-1 allele contributed to salt tolerance for one QTL on chromosome 4 (GRS8). Comparing our QTL with the published salt-tolerance QTL revealed that only one QTL overlapped with the previously reported QTL. GRS1 contains *RAS1* (Ren *et al.* 2010), previously identified as a causal gene for a QTL controlling variation in salt tolerance during early seedling development between Sha and Landsberg *erecta* (L*er*) (Ren *et al.* 2010). Recently, it was shown to be a microProtein that regulates the TGA1 transcription factor (Magnani *et al.* 2014) (Table 2).

**Table 2:** Comparison of GRS QTL for Tsu-1 × Sha mapped in this study and salt tolerance QTL reported in the literature

Chromosome	Name	Extrapolated Position of GRS, cM	Overlapping Salinity Tolerance QTL Peaks in the Literature
1	GRS1	12.7–16.9	Ler × Sha and Bay × Sha (Ren *et al.* 2010)
			Bay × Sha (Vallejo *et al.* 2010)
			Ler × Sha (Clerkx *et al.* 2004)
1	GRS2	36.9−41.1	None
1	GRS3	53.7−56.6	None
1	GRS4	62.2−73.5	None
1	GRS5	92.8−100.4	None
2	GRS6	78.6−98.3	None
3	GRS7	16.1−18.3	None
4	GRS8	29.3−36.8	None
5	GRS9	23.2−27.5	None

GRS, greening response to salt; QTL, quantitative trait locus.

**Table 3:** Comparison of SSQ QTL mapped for Tsu-1 × Sha in this study and the L*er* × Cvi QTL described by Alonso Blanco *et al.* (1999)

Chromosome	Name	Extrapolated Position of SSQ, cM	L*er* ×Cvi QTL position in cM (Effect Size)
1	SSQ1	6.5−15.3	**3−11 (10–15%)**
1	SSQ2	28.5−36.5	None
1	SSQ3	46.3−53.1	None
1	SSQ4	63.4−73.5	None
1	SSQ5	88.7−100.4	79−90 (5–10%)
1	SSQ6	111.3−116.6	None
1	SSQ7	122.4−127.3	None
2	SSQ8	75.5−82.7	None
4	SSQ9	73.3−78.1	**65−79 (<5%)**
5	SSQ10	66.5−80.0	**65−79 (<5%)**
5	SSQ11	84.5−97.5	**91−104 (5–10%)**
5	SSQ12	100.8−114.6	None
1			**20−29 (5–10%)**
3			**0−7 (5–10%)**
3			31−39 (5–10%)
4			**43−57 (<5%)**
5			42−55 (5–10%)

Bold-faced intervals are those QTLs from Alonso Blanco *et al.* (1999) that overlapped with Moore’s mapping results (Moore *et al.* 2013). SSQ, seed size QTL; QTL, quantitative trait locus.

For the seed size trait, even though maternal effects were observed (Table 1), no QTL showed parent-of-origin effects. Although seeds size QTL were reported previously (Alonso-Blanco *et al.* 1999), this study used different accessions. Nonetheless five SSQs overlapped with previously identified QTL (Table 3). Recently the QTL intervals reported by Alonso-Blanco *et al.* (1999) were refined (Moore *et al.* 2013), but because the genetic markers used for this study have not been placed on the combined genetic and physical maps, we cannot determine whether the recently refined peaks overlap with the SSQ intervals. Moore *et al.* (2013) analyzed three L*er* × Cvi mapping populations and reported that some of these QTL overlapped with those found by Alonso-Blanco (bold-face intervals in Table 3). The differences between these four existing L*er* × Cvi mapping populations were attributed to genotype × environment interactions that were revealed when plants were grown under different environmental conditions (Moore *et al.* 2013). The interval for SSQ1 corresponds to a previously reported QTL that accounts for 10–15% of the variation in seed size (Table 3). SSQ5, SSQ9, SSQ10, and SSQ11 overlap with previously reported QTL, each of which accounts for less than 5% or 5–10% of the variability (Table 3). SSQ2, SSQ3, SSQ4, SSQ6, SSQ7, SSQ8, and SSQ12 are novel QTL. Alonso-Blanco *et al.* (1999) found two seed size QTL on chromosome 3, but these were not identified in this study. This could be a problem of detection power, but more likely it is a difference in alleles among the different accessions or epistatic interactions that permitted loci to be found in RIL lines, but not in segregating F2 populations.

### Assessing the effect of sequencing depth on QTL detection power

**Figure 5:**
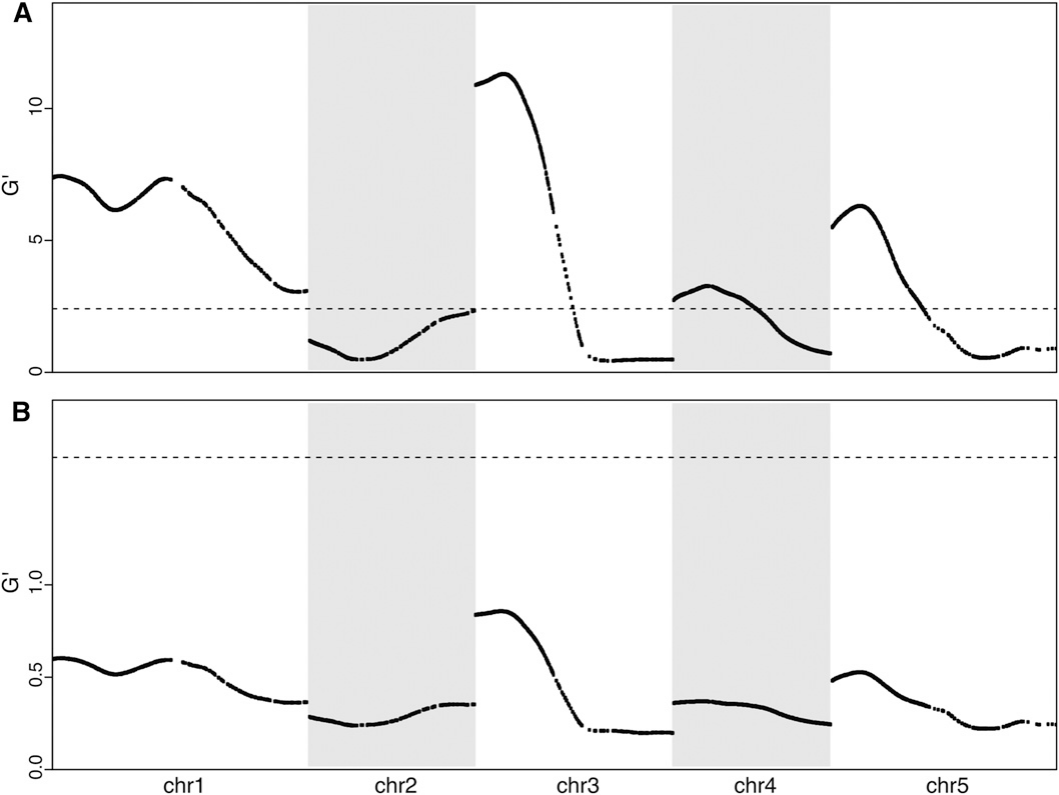
Simulated mapping derived from subsampling the empirical target-enriched extreme QTL mapping results of *Arabidopsis thaliana* seedling greening in response to salt. (A) Simulated mapping result for sequencing depth of 500×. (B) Simulated mapping result for sequencing depth 50×. Dashed horizontal lines are 1% false-discovery rate threshold for G′ values.

Using the empirically derived sequence data, we evaluated the effect of sequencing depth on QTL detectability for the greening in response to salt trait. We iteratively sampled 10- and 100-fold fewer reads at random (1000 iterations for each sequencing depth). Reducing the sequencing depth by 10-fold (500× sequencing depth) resulted in a QTL map similar to the one with 5000× sequencing depth, except for GRS6. For a trait with a heritability of 0.94, we expect a sequencing depth of 500× to detect a QTL of at least 5% effect ~70% of the time (Figure 1E). Therefore, the observed detection power (8/9 QTL) is consistent with the predictions. Reducing the sequencing depth by 100-fold (50X sequencing depth) yielded no significant peaks (Figure 5B). The *in silico* sampling experiments demonstrate the necessity of high sequencing depth for increasing QTL detection in bulked segregant analysis.

## Discussion

Here we developed a QTL mapping approach called TEX-QTL analysis, which increases the QTL detection power by incorporating deep sequencing of target-enriched SNP markers with bulked segregant analysis. Although bulked segregant analysis with large population size (X-QTL mapping) can identify many QTL with small effect sizes and additive effects (Lai *et al.* 2007; Ehrenreich *et al.* 2010), approaches that can increase the detection power of X-QTL have not been explored fully. To date, X-QTL mapping mainly has used whole-genome sequencing or SNP arrays to capture allelic frequency differences of SNP markers (Ehrenreich *et al.* 2010; Magwene *et al.* 2011; Yang *et al.* 2013; Pais *et al.* 2014). SNP arrays are limited by the number of SNP markers that could be spotted on the array. In contrast, whole-genome sequencing detects a majority of the SNPs in the genome but usually has relatively low sequencing depth per SNP marker to be cost-effective. More recently, exome capture was used to reduce the representation of genome sequencing for X-QTL mapping with relatively low sequencing depth (95−366×) (Chevalier *et al.* 2014). In theory, target enrichment and deep sequencing of SNP markers should permit the high sequencing depth needed for increased detection power. Our simulations suggested that by combining a large F2 population size, deeply sequenced markers, and 10–20% bulk size, we could in theory identify most QTL within two generations (Figure 1, Figure S1, and File S1). We empirically validated these theoretical predictions on two traits in *Arabidopsis thaliana*: 1) seed size and 2) seedling greening in response to salt.

Both our empirical and simulation results indicated increased detection power with TEX-QTL mapping. By sampling subpopulations of the sequence reads, we demonstrated that the higher detection power can be achieved with increased sequencing depth (Figure 5). Despite the reported multiple QTL mapping studies with different natural accessions on salt tolerance (Quesada *et al.* 2002; Clerkx *et al.* 2004; Rus *et al.* 2006; Galpaz and Reymond 2010; Ren *et al.* 2010; Vallejo *et al.* 2010; Derose-Wilson and Gaut 2011; Roy *et al.* 2013), this study detected several new QTL peaks. These newly identified QTL might be due to an increased QTL mapping power using this TEX-QTL approach. It is also possible that the new QTL might reflect the unique genetic components contributing to the phenotypic variation of the mapping population derived from Sha × Tsu-1. Comparing TEX-QTL and traditional RIL-based QTL mapping by using populations derived from same parental lines should help address this issue in the future.

We developed a statistical pipeline to identify TEX-QTL peaks along with interval estimates of their locations (File S6). In the framework of whole-genome sequencing-based bulked segregant analysis, several statistical methods have been developed recently, including the smoothed G test (Magwene *et al.* 2011), a dynamic Bayesian network (Edwards and Gifford 2012), and a Hidden Markov Model (Duitama *et al.* 2014). Application of the latter two methods to TEX-QTL mapping data yielded poorly resolved QTL regions (data not shown), indicating that these approaches might not be suitable for TEX-QTL mapping. The smoothed G test uses neighboring SNPs to reduce the random noises associated with the sequencing reads, but doesn’t identify putative QTL peaks and their support intervals. Therefore, we developed and applied a model-based method, which resolved linked QTL regions into more discrete peaks (Figure 3).

One limitation of the current method is the detection of closely linked QTL. As suggested in the simulation, two very closely linked QTL may be identified as one QTL. Because the support intervals are calculated under the assumption of one causal gene, and the center of the interval would be positioned between two closely linked QTL, the interval may not include both causal genes. It may be helpful to examine the loci flanking outside of the support interval if no causal gene can be found within the interval.

Another limitation of TEX-QTL mapping, similar to that of bulked segregant analysis in general, is that the allele frequency of the bulks does not permit the detection of epistasis between QTL or calculation of the contribution of each QTL to the phenotypic variation. Nevertheless, TEX-QTL mapping is advantageous in that it offers a quick and cost-effective way to identify a theoretically maximal number of QTL (QTL with both small and large effects) contributing to the variation of a trait.

Here we demonstrated that TEX-QTL mapping increases the resolution of complex traits rapidly and reveals genetic architectures in a cost-effective manner. TEX-QTL mapping is applicable to many species such as insects, fish, frogs, or any other organisms where genetic crosses can be made and large F2 populations can be generated. It is particularly well suited to species with large genomes, for which obtaining the high sequencing depth needed to achieve increased QTL detection power is not practical with whole genome sequencing. This approach could accelerate the discovery of genetic architectures underlying complex traits and provides a powerful way to harness the genetic potential of germplasm to increase crop or livestock performance through marker-assisted breeding or genetic engineering.

## 
